# Production of d-glucaric acid with phosphoglucose isomerase-deficient *Saccharomyces cerevisiae*

**DOI:** 10.1007/s10529-023-03443-2

**Published:** 2023-12-08

**Authors:** Mervi Toivari, Maija-Leena Vehkomäki, Laura Ruohonen, Merja Penttilä, Marilyn G. Wiebe

**Affiliations:** VTT Technical Research Centre of Finland Ltd, Tekniikantie 21, P.O. Box 1000, 02044 Espoo, Finland

**Keywords:** Glucaric acid, Glucarate, Metabolic engineering, Myo-inositol, Phosphoglucose isomerase, *Saccharomyces cerevisiae*

## Abstract

d-Glucaric acid is a potential biobased platform chemical. Previously mainly *Escherichia coli,* but also the yeast *Saccharomyces cerevisiae,* and *Pichia pastoris,* have been engineered for conversion of d-glucose to d-glucaric acid via myo-inositol. One reason for low yields from the yeast strains is the strong flux towards glycolysis. Thus, to decrease the flux of d-glucose to biomass, and to increase d-glucaric acid yield, the four step d-glucaric acid pathway was introduced into a phosphoglucose isomerase deficient (Pgi1p-deficient) *Saccharomyces cerevisiae* strain. High d-glucose concentrations are toxic to the Pgi1p-deficient strains, so various feeding strategies and use of polymeric substrates were studied. Uniformly labelled ^13^C-glucose confirmed conversion of d-glucose to d-glucaric acid. In batch bioreactor cultures with pulsed d-fructose and ethanol provision 1.3 g d-glucaric acid L^−1^ was produced. The d-glucaric acid titer (0.71 g d-glucaric acid L^−1^) was lower in nitrogen limited conditions, but the yield, 0.23 g d-glucaric acid [g d-glucose consumed]^−1^, was among the highest that has so far been reported from yeast. Accumulation of myo-inositol indicated that myo-inositol oxygenase activity was limiting, and that there would be potential to even higher yield. The Pgi1p-deficiency in *S. cerevisiae* provides an approach that in combination with other reported modifications and bioprocess strategies would promote the development of high yield d-glucaric acid yeast strains.

## Introduction

d-Glucaric acid (d-saccharic acid) is a di-carboxylic acid that can be used for example to produce furan dicarboxylic acid (van Strien et al. 2020) or various polyamides, and polyesters (Sakuta and Nakamura 2019). Biotechnical conversion of d-glucose to d-glucaric acid (or to the conjugate salt d-glucarate) can provide a selective and less energy intensive alternative to chemical production processes (Zhang et al. 2021).

Moon et al. (2009) were the first to engineer *Escherichia coli* for production of d-glucaric acid. They introduced activities for myo-inositol-1-phosphate synthase, myo-inositol oxygenase and uronate dehydrogenase into *E. coli* for conversion of D-glucose via glucose-6-phosphate and 1L-myo-inositol-1-phosphate (1 d-myo-inositol 3-phosphate) to myo-inositol, d-glucuronate and finally to d-glucaric acid, resulting in production of 1.1 g L^−1^ of d-glucaric acid (Table 1). By introducing a polypeptide scaffold to co-localize the pathway enzymes d-glucaric acid concentration was increased to ~ 2.5 g L^−1^ (Moon et al. 2010). The myo-inositol oxygenase (MIOX) with its di-iron center and low activity was suggested to be the rate-limiting step (Moon et al. 2010). The MIOX activity was subsequently improved by using an ***N***-terminal fusion of small ubiquitin-related modifier (SUMO) to MIOX, showing 75% increase in myo-inositol to d-glucaric acid conversion (Shiue and Prather 2014). Overexpression of myo-inositol-1-phosphate phosphatase from *E. coli* was tested for further enhancement of the process and the flux of d-glucose from catabolism towards myo-inositol-1-phosphate was redirected by deletion of the phosphoglucose isomerase (Pgi) and glucose 6-phosphate dehydrogenase (Zwf) encoding genes in *E. coli* (Shiue et al. 2015). This resulted in an increased yield of d-glucaric acid from d-glucose (yield 0.73 g g^−1^ with titer of 1.19 g L^−1^, d-xylose as supplementing carbon source). Another approach used to decrease the flux to glycolysis was altering Pfk activity (Brockman and Prather 2015; Gupta et al. 2017; Hou et al. 2020). The d-glucaric acid pathway has also been used as a demonstration pathway for different synthetic biology approaches including use of MAGE (Raman et al. 2014), and small molecule reporter (Rogers and Church 2016). Dynamic pathway regulation with a quorum sensing based system or myo-inositol biosensor (Doong et al. 2018; Verma et al. 2022), regulation of Pgi translation by a d-fructose dependent control system (Qu et al. 2018), and NAD + regeneration system (Su et al. 2020) have been applied for d-glucaric acid production in *E. coli*. d-Glucaric acid production has also been demonstrated by in vitro conversion (Lee et al. 2016; Petroll et al. 2020; Su et al. 2019). Without myo-inositol addition, volumetric titers have remained between 1 and 2.5 g L^−1^, although a recent study reported 5.35 g L^−1^ for intra plus extracellular titer in *E. coli* (Su et al. 2020) (reviewed by (Chen et al. 2020)).

**Table 1:** Production of d-glucaric acid by *E. coli*, yeast *S. cerevisiae* or *P. pastoris* in vivo. The highest extracellular volumetric titer and corresponding yield of a study are presented. Partly adapted from (Chen et al. 2020)

Organism	Year	Pgip modified (yes/no)	Culture type	Carbon source	Titer (g L^−1^)	Yield (g g^−1^ d-glucose)	References
*E. coli*	2009	No	Batch (flask)	d-glucose	1.13	0.151^a^	Moon et al. (2009)
*E. coli*	2009	No	Batch (flask)	d-glucose	1.7	–	Dueber et al. (2009)
*E. coli*	2010	No	Batch (flask)	d-glucose	2.50	0.250	Moon et al. (2010)
*E. coli*	2014	No	Batch (flask)	Myo-inositol	4.85	0.449	Shiue and Prather (2014)
*E. coli*	2015	Yes	Batch (flask)	d-glucose	1.19	0.73	Shiue et al. (2015)
*E. coli*	2015	No	Simulated fed-batch, BioLector (starch release)	d-glucose/starch	1.56	0.124	Reizman et al. (2015)
*E. coli*	2017	No	Batch (flask)	d-glucose	~ 0.85	–	Gupta et al. (2017)
*E. coli*	2018	No	Batch (bioreactor)	d-glucose	1.98	0.229^a^	Doong et al. (2018)
*E. coli*	2018	Yes	Batch/sucrose (flask)	Sucrose	~ 1.42	0.27	Qu et al. (2018)
*E. coli*	2020	Yes	Batch (flask)	d-glucose	3.91	0.514^a^	Su et al. (2020)
*E. coli*	2020	No	Batch (bioreactor)	d-glucose	1.56	–	Hou et al. (2020)
*P. pastoris*	2016	No	Fed-batch (bioreactor)	d-glucose, myo-inositol	6.61	–	Liu et al. (2016)
*S. cerevisiae*	2016	No	Batch (flask)	d-glucose, myo-inositol	1.6	–	Gupta et al. (2016)
*S. cerevisiae*	2016	No	Batch with spiking glucose (flask)	d-glucose	0.98	0.033	Gupta et al. (2016)
*S. cerevisiae*	2018	No	Fed-batch (bioreactor)	d-glucose, myo-inositol	6.0	–	Chen et al. (2018)
*S. cerevisiae*	2020	No	Fed-batch (bioreactor)	d-glucose	5.23	–	Zhang et al. (2020)
*S. cerevisiae*	2020	No	Batch (flask)	d-glucose, myo-inositol	1.76	–	Marques et al. (2020)
*S. cerevisiae*	2021	No	Batch with spiking glucose (bioreactor)	d-glucose, myo-inositol	10.6	–	Zhao et al. (2021)
*S. cerevisiae*	2021	No	Fed-batch (flask)	d-glucose, myo-inositol	11.21	–	Li et al. (2021)
*S. cerevisiae*	2021	No	Fed-batch (flask)	d-glucose	4.52	–	Li et (al. (2021)
*S. cerevisiae, T. reesei*	2021	No	CBP (flask)	Cellulose (Avicel)	0.54	0.036	Li et al. (2021)
				Corn stover	0.45	0.03	
*S. cerevisiae*	2021	No	Batch (flask)	Galactose, myo-inositol	0.142	–	Cheah et al. (2021)
*S. cerevisiae*	2022	No	Fed-batch (flask)	d-glucose, myo-inositol	12.96	–	Fang et al. (2022)
*S. cerevisiae*	2022	No	Fed-batch (flask)	d-glucose	6.94	–	Fang et al. (2022)
*S. cerevisiae, T. reesei*	2022	No	CBP (flask)	Corn stover	6.42	–^b^	Fang et al. (2022)
*S. cerevisiae*	2022	No	Fed-batch (bioreactor)	d-glucose	9.5	0.216	Guo et al. (2022)
*S. cerevisiae, T. reesei*	2023	No	CBP (bioreactor)	Corn stover	10.03	–	Fang et al. (2023)
				Wheat straw	9.53		
				Rice straw	8.87		
				Switchgrass	10.66		

^**a**^Originally reported in mol/mol

^b^Reported only per steam exploded corn stover

Yeast are considered advantageous for organic acid production because of their low pH tolerance and robustness (Abbott et al. 2009). Yeasts have also been engineered for production of d-glucaric acid, although more recently than *E. coli* (Table 1). In 2016 Gupta et al. first engineered *Saccharomyces cerevisiae* for production of d-glucaric acid by expressing the d-glucaric acid pathway genes coding for inositol monophosphatase, myo-inositol-1-phosphate synthase, myo-inositol oxygenase either from *Mus musculus* or from *Arabidopsis thaliana* and uronate dehydrogenase in a *opi1* deletion background (Gupta et al. 2016). The engineered yeast produced a maximum titer of 0.56 g L^−1^ in batch culture, and 0.98 g L^−1^ in fed-batch. Liu et al. (2016) introduced the pathway into *Pichia pastoris* and produced 0.107 g L^−1^ d-glucaric acid from d-glucose. Since these pioneering studies further pathway engineering approaches by e.g. enzyme and expression optimization, use of scaffolds, or by improving viability, have increased d-glucaric acid titers up to 9.5 g L^−1^ (Fang et al. 2022, 2023; Li et al. 2021; Zhang et al. 2020). In general, co-feeding of myo-inositol has resulted in higher final d-glucaric acid titers, up to 12.96 g L^−1^ in *S. cerevisiae* (Chen et al. 2018; Fang et al. 2022; Guo et al. 2022; Gupta et al. 2016; Li et al. 2021; Marques et al. 2020; Zhao et al. 2021), and 6.61 g L^−1^ in *P. pastoris* (Liu et al. 2016). The reported yields on d-glucose using yeast are scarce, and typically below 0.1 g g^−1^, although recently a yield of 0.216 g g^−1^ (Guo et al. 2022) was reported (Table 1).

**Fig. 1:**
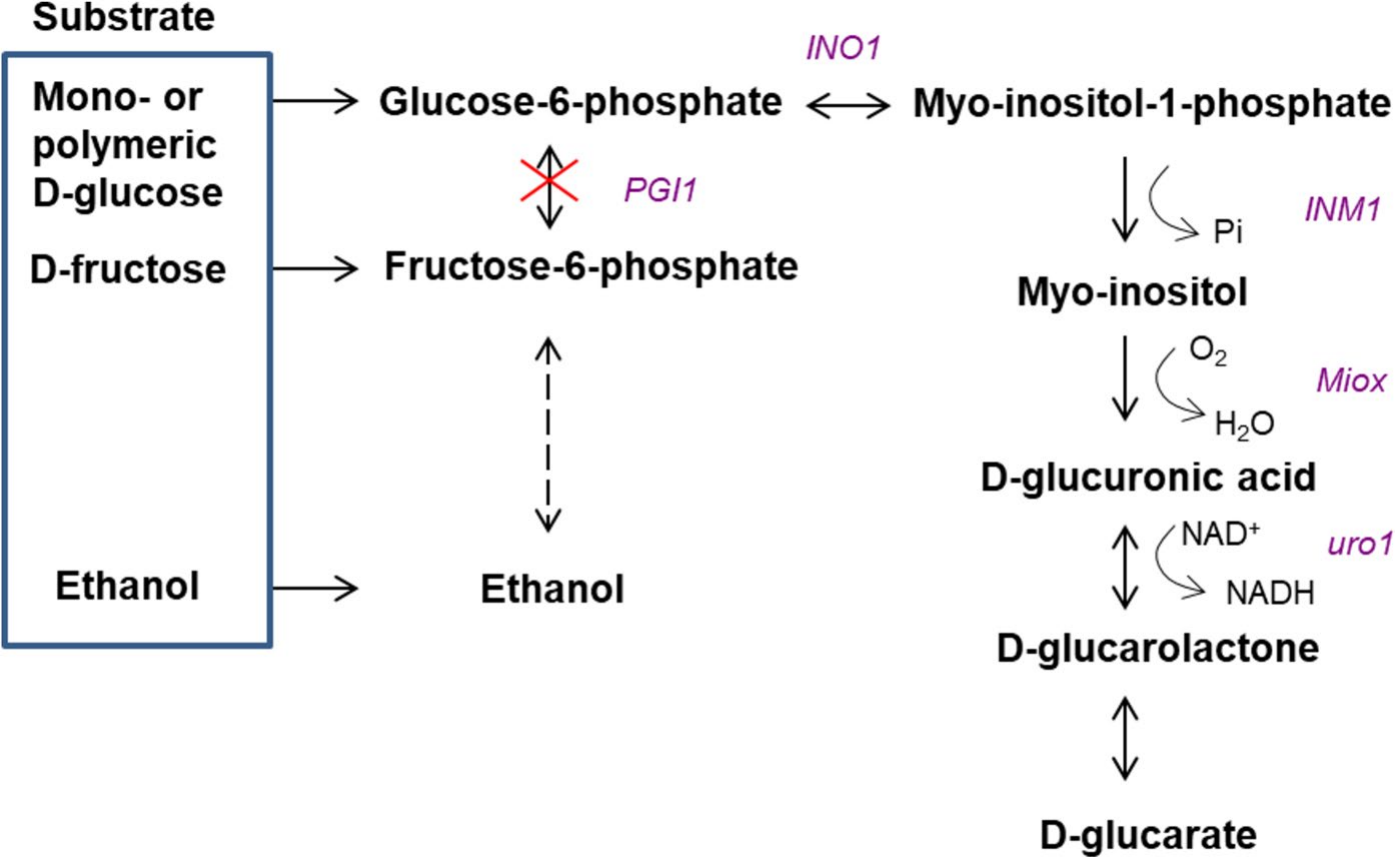
Schematic outline for conversion of d-glucose to d-glucaric acid with Pgi1p-deficient *S. cerevisiae*. In the Pgi1p-deficient strain, d-glucose should be channeled to myo-inositol, while d-fructose and ethanol would allow growth and support the production of biomass. Glucose-6-phosphate can also be channeled to pentose phosphate pathway or to storage carbohydrates

In *E. coli* deletion of the phosphoglucose isomerase (Pgi) and glucose 6-phosphate dehydrogenase (Zwf) encoding genes resulted in 2.9-fold (Qu et al. 2018), or nearly 18-fold higher yield on d-glucose (Shiue et al. 2015), compared to when the genes were present. The phosphoglucose isomerase encoding gene has not been deleted from *S. cerevisiae* or *P. pastoris* strains engineered for d-glucaric acid production. The phosphoglucose isomerase (Pgi1p) -deficient *S. cerevisiae* strains metabolize d-glucose only poorly, whereas the Pgi-deficient strains of *E. coli* are able to grow on d-glucose (Vinopal et al. 1975). Even relatively low (< 2 g L^−1^) d-glucose concentrations inhibit or reduce the growth of *S. cerevisiae* Pgi1p-deficient strains, possibly because of ATP depletion or glucose-6-phosphate accumulation (Maitra 1971). The accumulation of glucose-6-phosphate, the first metabolite in the d-glucaric acid pathway, could be advantageous for directing d-glucose flux from glycolysis to d-glucaric acid and thus improve the yield of d-glucaric acid on d-glucose. We introduced the d-glucaric acid pathway into a Pgi1p-deficient *S. cerevisiae* strain (Fig. 1) and studied d-glucaric acid production from monomeric and polymeric d-glucose substrates in shaken flasks and controlled bioreactor conditions with varying nitrogen concentrations. The formation of myo-inositol and d-glucaric acid, and the absence of d-glucuronate was confirmed by using ^13^C-labelled d-glucose and GC–MS analysis.

## Materials and methods

### Strains and strain construction

*E. coli* strains DH5alpha or TOP10 were used for cloning steps, plasmid propagation and storage, and the *Saccharomyces cerevisiae* strain FY834 for recombination cloning. *S. cerevisiae* CEN.PK2-1D (VW-1B; *MATα, leu2-3/112 ura3-52 trp1-289 his3∆1 MAL2-8*^*c*^* SUC2*) renamed H1346, and the *pgi1*-deficient strain H2493, described previously (Verho et al. 2002) were the parental strains in which the d-glucaric acid biosynthetic pathway was expressed.

Strain H2493 was cured from tryptophan and/or leucine auxotrophies by transforming it with PCR fragments amplified with primers BCoreMT_LEU2_F and BCoreMT_LEU2_R or BCoreMT_TRP1_F and BCoreMT_TRP1_R (Table 2), using genomic DNA from *S. cerevisiae* strain S288C as a template, and selecting colonies on plates lacking leucine and/or tryptophan, as appropriate.

**Table 2:** Primers used for strain construction

Name	Sequence
BCoreMT_LEU2_F	ATGTCTGCCCCTAAGAAGATC
BCoreMT_LEU2_R	TTAAGCAAGGATTTTCTTAAC
BCoreMT_TRP1_F	ATGTCTGTTATTAATTTCAC
BCoreMT_TRP1_R	CTATTTCTTAGCATTTTTGACG
II_IMP_chr8_BamHI/EcoRI/F	AGGATCCGAATTCACTATGACCATTGATCTAGCTTC
II_IMP_chr8_Bam/R	CGGATCCTCAGTCATATTTCAAATGGCCATC
BcoreMT_MIOX_F	TAGCAATCTAATCTAAGTTTTAATTACAAACTCGAGTAAAATGAAAGTTGATGTTGGTCC
BcoreMT_MIOX_R	CCAAACCTCTGGCGAAGAAGTCCAAAGCTGTCGACTCACCAAGACAAAGTACCTG
BcoreMT_URO1_F	ATCTATAACTACAAAAAACACATACATAACCCGGGAAAATGGCGATGAAACGGCTTC
BcoreMT_URO1_R	TTTAATTTGCGGCCGGTACCCAATTCGCCGGATCCTCAGCTCTGTTTGAAGATCG
BcoreMT_INO1_F	CTTTTTACAACAAATATAAAACCAAAAGCGGCCGCAAAATGACAGAAGATAATATTGC
BcoreMT_INO1_R	GCACCACCACCAGTAGAGACATGGGAGATCCCGCGGTTACAACAATCTCTCTTCGA
S.c GRE3 nt-5frw	GGCCGCGGCCGCCAGCTGTAAAGATGTCTTCACTGGTTACTC
S.c GRE3 nt310rev	CCGGCGTACGAAGCTTGGATCCAATCAAGTCCCATATCGCTTAAGG3
S.c GRE3 nt677frw	GGCCGATATCTCATTGAGATGGACTTACAGTTGG
S.c GRE3 nt966rev	CCGGACTAGTTTTACCATCCAACCAGGTCCATGG

The myo-inositol oxygenase encoding gene *Miox* from Mouse was obtained as a synthetic gene, codon optimized for *S. cerevisiae* (Gene Art, Germany). *S. cerevisiae* genomic DNA (from S288C or CEN.PK2-1D) was used to amplify the *INO1* (YJL153C) and *INM1* (YHR046C) genes (resulting amino acid sequences are the same in both genomes). The uronate dehydrogenase (Udh) gene of *Agrobacterium tumefaciens* (*uro1*, GI:223,717,948) (Yoon et al. 2009; Boer et al. 2010) was obtained from the plasmid described by Boer et al. (2010). *Miox*, *INO1* and *uro1* were cloned into pRS426 based (B712, (Christianson et al. 1992)) plasmid B5054 (Salusjärvi et al. 2017) that has three promoter-terminator pairs *pTEF1* and t*ADH1*, *pTPI1* and *tCYC1*, *pPGK1* and *tPGK1* by homologous recombination using primers BcoreMT_MIOX_F and BcoreMT_MIOX_R for *Miox*, BcoreMT_INO1_F and BcoreMT_INO1_R for *INO1*, and BcoreMT_URO1_F and BcoreMT_URO1_R for *uro1* (Table 2), resulting in plasmid B5310. The *INM1* gene was expressed either as a single copy genomic integrant or from a multicopy plasmid. *INM1* was amplified with primers II_IMP_chr8_BamHI/EcoRI/F and II_IMP_chr8_Bam/R, digested with BamHI and cloned between the *PGK1* promoter and terminator in a YEplac195-based multicopy vector B1181 (Toivari et al. 2010a) digested with BglII to create plasmid INM1-B1181. The *pPGK1*-*INM1*-*tPGK1* cassette was subsequently moved to YEplac181 (B548, (Gietz and Sugino 1988)) by releasing the cassette with *HindI*II and ligating it to the *Hind*III site of YEplac181 resulting in plasmid B5154. For genomic integration the *INM1* was targeted to *GRE3* locus. The 315 bp and and 290 bp regions of *GRE3* gene were amplified from the H1346 genomic DNA with primer pairs S.c GRE3 nt-5frw and S.c GRE3 nt310rev and S.c GRE3 nt677frw and S.c GRE3 nt966rev, respectively, where the numbers are relative to nucleotide A in ATG of the *GRE3* gene. The *BamH*I site for cloning the gene expression cassette was included in the 315 bp region. The 315 bp region was ligated into a *Pvu*II and *BsiW*I linearised plasmid pUG6 (Gueldener et al. 2002), and the plasmid obtained was then cut with *EcoR*V and *Spe*I for introducing the 290 bp region. The resulting plasmid, containing the KanMX cassette flanked by the *S.cerevisiae GRE3* regions, was named pMLV84. The pMLV84 was digested with *BamH*I and the *pPGK1*-*INM1*-*tPGK1* cassette digested with *Hind*III from INM1-B1181, both fragments were blunt-ended and ligated resulting in INM1-pMLV84. The integration cassette, released with *Not*I, was transformed into the *pgi1*-deficient strain cured for TRP and LEU. A cassette without *INM1* was transformed to create a control strain.

The *INO1*-*Miox*-*uro1* B5310 plasmid was introduced to the parental strain H1346 (with intact *PGI1*) with or without the *INM1* plasmid B5154, resulting in strains H4254 and H4355, respectively. The *INM1* was integrated to the *GRE3* locus of H1346 as described for the *pgi1* strains, and the plasmid B5310 was introduced, resulting in strain H5156. The *INO1*-*Miox*-*uro1* plasmid B5310 was introduced to the *pgi1*-deficient strain cured with *TRP1* and *LEU2*, with or without *INM1* integration, resulting in strains H4350 and H4344, respectively. The B5310 plasmid was also introduced to the *pgi1*-deficient strain cured for TRP, together with the *INM1* plasmid B5154, resulting in strain H4346 or to *pgi1*-deficient strain with KMX integrated into *GRE3*, resulting in strain H4351. Corresponding control strains with empty vectors B712 + B548 H4345, and H4349 were also created. The final strains used in the study are listed in Table 3.

**Table 3:** Yeast strains used in the study

Strain	Relevant genomic modifications	Pathway genes overexpressed	Plasmid(s)
H4354	–	*INO1, Miox, uro1*	B5310
H4355	–	*INO1, Miox, uro1; INM1*	B5310 + B5154
H4356	*gre3::INM1*	*INO1, Miox, uro1; INM1int*	B5310
H4344	*pgi1*Δ	*INO1, Miox, uro1*	B5310
H4345	*pgi1*Δ	–	B712
H4346	*pgi1*Δ	*INO1, Miox, uro1; INM1*	B5310 + B5154
H4349	*pgi1*Δ	–	B712 + B548
H4350	*pgi1*Δ, *GRE3::INM1*	*INO1, Miox, uro1; INM1int*	B5310
H4351	*pgi1*Δ, *GRE3::KMX*	*INO1, Miox, uro1*	B5310

### Media and culture conditions

Yeast strains were cultured in 20 or 50 mL volume on modified synthetic complete medium (YSC, (Sherman et al. 1983)) without uracil and/or leucine, with 2% (w/v) d-glucose or the d-glucose and d-fructose concentrations indicated in the results, in 100 or 250 mL Erlenmeyer flasks, respectively, at 250 rpm, 30 °C. The *pgi1*-deficient strains were grown with 0.5 g d-glucose L^−1^ and 20 g d-fructose L^−1^ as carbon source for maintenance and biomass generation. The (NH_4_)_2_SO_4_ concentration in YSC was reduced from 5 to 1.5 g L^−1^ for nitrogen-restricted batch cultures. The flask cultures with *pgi1*-deficient strains were buffered by CaCO_3_ (1%, w/v). For slow glucose release, EnBase B (Biosilta, Oulu, Finland) was prepared according to manufacturer’s instructions, except pH was adjusted to pH 5.6 with HCl and enzyme (5 µL to 20 mL culture broth) added after 24 h incubation, because d-glucose (~ 1 g L^−1^) was present in the prepared medium. Cellulose (α-cellulose, Sigma) was provided as 16.6 g L^−1^, with 4.5 mL Cellulast 1.5 L added to a volume of 250 mL in bioreactor culture.

For larger scale cultures, yeast were grown in 250 to 500 mL medium (YSC-ura, or YSC-ura-leu) in Multifors bioreactors (max. working volume 500 mL, Infors HT, Switzerland) at pH 5.5, 30 °C, 1 volume air [volume culture]^−1^ min^−1^ (vvm) and 500 rpm agitation with 2 marine impellors, as previously described (Toivari et al. 2010a, b). The pH was maintained constant by addition of 2 M NaOH or 1 M H_2_PO_4_. Clerol antifoaming agent (Cognis, France, 0.08–0.10 µL^−1^) was added to prevent foam formation. d-Glucose concentration in the culture supernatant was monitored by HPLC and d-glucose, d-fructose and/or ethanol were added as pulses to keep the d-glucose concentration between 0.5 and 5 g L^−1^, while providing d-fructose or ethanol as an energy and carbon source for growth.

Biomass was measured as optical density (OD) at 600 nm (OD_600_) or as dry weight. For dry weight, samples were collected in 2 mL pre-dried, pre-weighed microcentrifuge tubes, washed twice with equal volume distilled water and dried at 105 °C.

The number of metabolically active (vital) cells was determined microscopically by methylene blue (0.25 g L^−1^ in 0.04 M NaCitrate buffer pH 8.3) staining. For the purpose of clarity the metabolically active cells will be referred to as viable and the inactive as metabolically inactive cells. Both empty and stained cell were counted as metabolically inactive cells.

### Chemical analyses

For determination of intracellular d-glucaric acid concentration, cells were collected from 10 mL culture. Cell pellets were washed with 1.8 mL of 0.9% w/v NaCl solution (9 g L^−1^), and then 1.8 mL deionised water, and frozen at − 20 °C to disrupt membranes. The frozen pellets were freeze-dried using a Christ Alpha 2–4 lyophiliser (Biotech international, Belgium), removing all excess moisture. Intracellular d-glucaric acid was extracted from the lyophilized pellets (6 to 45 mg biomass) in 5 mM H_2_SO_4_ (0.5 mL) as described by Nygård et al. (2011) for extraction of d-xylonate. Cell debris was removed by centrifugation and the supernatant analysed by GC–MS. Intracellular concentrations are given as mg per g dry biomass. For a conservative estimate of intracellular concentration, assume that 1 g dry cell weight corresponds to 2 mL cell volume (de Koning and van Dam 1992; Gancedo and Serrano 1989). This estimate is conservative since it does not take into account the volume of intracellular organelles, variation in cell wall thickness, or the contribution of dead cells to the dry biomass.

**Fig. 2:**
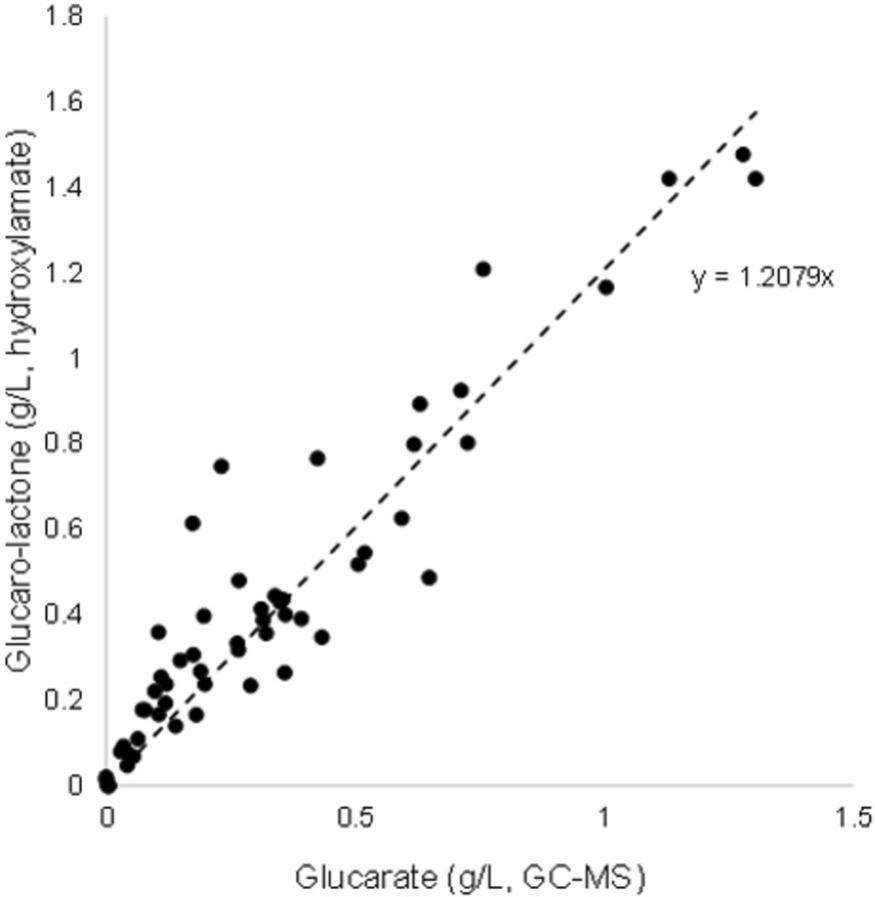
Correlation between d-glucaric acid measured by GC–MS and the lactone measured using the hydroxylamate assay, with background subtracted

Concentrations of d-glucose and d-fructose, ethanol, glycerol, and acetate, were analysed by HPLC using a Fast Acid Analysis Column (100 mm × 7.8 mm, BioRad Laboratories, Hercules, CA) linked to an Aminex HPX-87H column (BioRad Labs, USA) with 5 mM H_2_SO_4_ as eluent and a flow rate of 0.3 mL min^−1^. The column was maintained at 55 °C. Peaks were detected using a Waters 410 differential refractometer and a Waters 2487 dual wavelength UV (210 nm) detector. The ability of d-glucaric acid and its dissociated form d-glucarate to form glucaric acid 1,4-lactone or dilactone needs to be considered in the analytics. d-Glucaric acid, glucaric acid 1,4-lactone, d-glucuronate and myo-inositol were quantified with GC–MS. The d-glucaric acid concentration is presented as the sum of d-glucaric acid and glucaric acid 1,4-lactone. Samples (100 µL), with arabitol as internal standard, were evaporated to dryness and silylated by adding 100 µL pyridine, 100 µL chlorotrimethylsilane and 100 µL N,O-Bis(trimethylsilyl)trifluoroacetamide (BSTFA). Derivatisation was performed at + 70 °C for 60 min. Derivatised samples (1 µL) were subjected to GC–MS analysis (Agilent 6890 Series, USA combined with Agilent, 5973 Network MSD, USA and Combipal injector, Varian Inc., USA). Analytes were injected on split mode (30:1) (200 °C) and separated on a ZB-1HT INFERNO capillary column (30 m × 0.25 mm) with a phase thickness 0.25 µm (Phenomenex, Denmark). Helium (0.9 mL min^−1^) was used as carrier gas in constant flow mode. The temperature program started at 70 °C with 3 min holding time and then increased 10 °C min^−1^ up to 320 °C. Mass selective detector (MSD) was operated in electron-impact mode at 70 eV, in the full scan m/z 40–550. The ion source temperature was 250 °C and the interface was 280 °C. Compounds were identified according to corresponding standards and with the Palisade Complete 600 K Mass spectral library (Palisade Mass Spectrometry, USA).

d-Glucaric acid concentrations were also measured as the lactone using the hydroxymate method (Lien 1959) as described by Toivari et al. (2010b). The lactone assay was used for analysing samples from cultures grown with cellulose and although it correlated well with GC–MS results (Fig. 2), the assay would also measure d-gluconic acid, which was probably present in the sample. Thus, lactone measurements should be considered as indicative, but may be over-estimates.

## Results

### d-Glucose conversion to d-glucaric acid with *pgi1-*deficient *S. cerevisiae*

The *pgi1*-deficient *S. cerevisiae* CEN.PK2-1D strains expressed *INO1*, *Miox* and *uro1* from a multicopy plasmid and *INM1* either as the native genomic copy (H4344), from an additional integrated copy under a constitutive promoter (H4350), or from a multicopy plasmid (H4346) (Table 3). Strains H4349 or H4345 without the glucarate pathway were used as controls. Similar strains, with the d-glucaric acid pathway, but with intact *PGI1* gene (H4354, H4355, H4356), were also created (Table 3).

The strains with intact *PGI1* gene were grown in flask cultures with 2% (w/v) d-glucose as a sole carbon source. In 48 h at most 35 mg d-glucaric acid L^−1^ was produced (data not shown).

Although d-glucose inhibits the growth of *pgi1*-deficient strains at concentrations above 2 g L^−1^, concentrations up to 4 or 5 g L^−1^ could be tolerated in cultures with sufficient biomass (OD600 12). With 4 g d-glucose L^−1^ and 20 g d-fructose L^−1^, strain H4344, with the d-glucaric acid pathway with endogenous *INM1*, produced 240 ± 11 mg d-glucaric acid L^−1^ in 98 h, whereas the control strain H4345 did not produce any d-glucaric acid. In addition, 370 ± 11 mg L^−1^ of myo-inositol was detected from strain H4344 compared to 100 ± 26 mg L^−1^ from H4345. All d-glucose and d-fructose were consumed, biomass, ethanol, acetate and glycerol were formed and partly consumed.

The other *pgi1*-deficient *S. cerevisiae* strains H4346, with the d-glucaric acid pathway with *INM1* on a multicopy plasmid, and the control strain H4349 were cultivated in the presence of 3.4 g L^−1^ uniformly labelled d-glucose. To decrease biomass formation, lower d-fructose (12 g L^−1^) and ammonium sulphate concentrations were used with initial high biomass (OD600 13). In 73 h, 280 mg L^−1^ of labelled d-glucaric acid and 330 mg L^−1^ labelled myo-inositol was detected. Thus, altogether, 610 mg L^−1^ of ^13^C-labelled d-glucose was directed to the d-glucaric acid pathway confirming formation of d-glucaric acid from d-glucose via myo-inositol. The d-glucaric acid pathway intermediate d-glucuronate was not detected. With the control strain H4349 no d-glucaric acid, or d-glucuronate, was detected. With higher d-glucose concentration the *pgi1*-deficient strains lost viability (data not shown).

### Evaluation of different feeding strategies for production of d-glucaric acid in bioreactor cultures

**Fig. 3:**
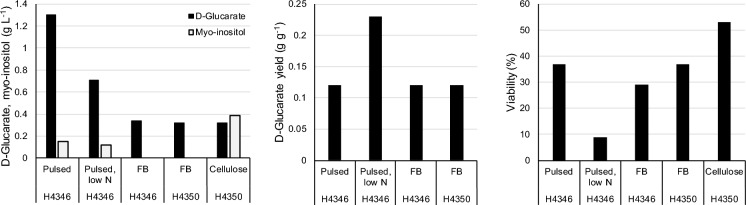
d-Glucaric acid and myo-inositol concentrations (left), d-glucaric acid yield on glucose (middle) and the proportion of viable cells (right) in the culture after 70 h cultivation for two d-glucaric acid producing strains (H4346 and H4350). Cultures were grown in bioreactors at pH 5.5 with pulsed addition of d-glucose (with d-fructose or ethanol for energy) or in fed-batch culture (FB) or a batch culture with cellulose as the source of d-glucose. The nitrogen supply was reduced in one culture receiving pulses of d-glucose. Maximum d-glucaric acid titers were observed after 160, 160, 117, 117 and 191 h for the 5 cultures shown. Yield was not determined for the cellulose batch culture

**Fig. 4:**
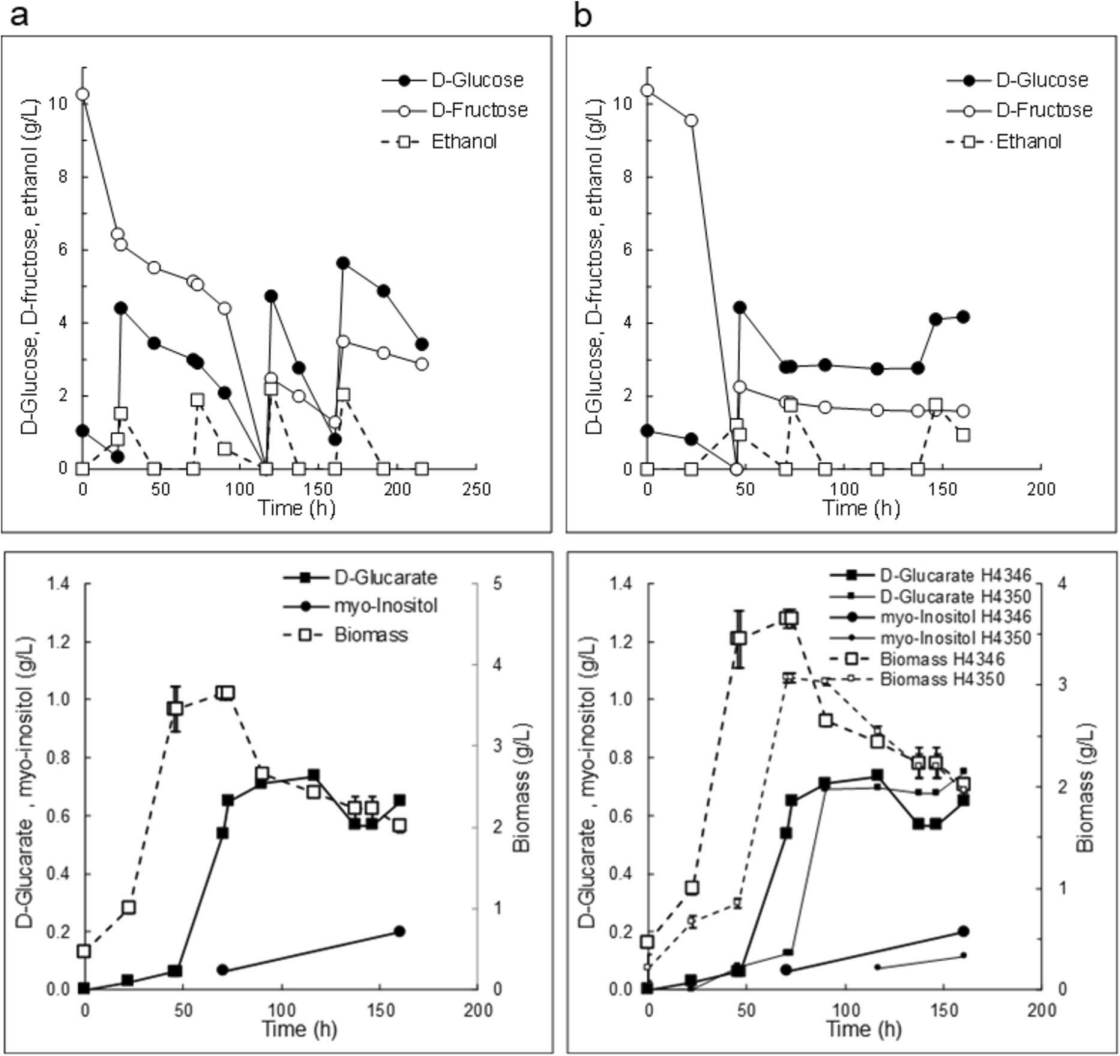
*S. cerevisiae pgi1*-deficient strain with a pathway for conversion of d-glucose to d-glucaric acid (pathway genes expressed on multicopy plasmids, H4346), was grown in SC -ura -leu **a** nitrogen sufficient or **b** nitrogen-restricted medium at pH 5.5 with d-glucose, d-fructose and ethanol as carbon sources (upper graphs), added initially and as pulses at intervals, as shown. Lower graphs show the concentrations of d-glucaric acid, myo-inositol and biomass produced. Data on product formation by a strain with *INM1* integrated (H4350) are also shown (small symbols) for nitrogen-restricted medium. Substrate concentrations and feeding were similar for H4350 and H4346 and are only shown for H4346 on the upper graph. Error bars (biomass) represent ± sem (n = 3) and where not visible were smaller than the symbol

The *pgi1*-deficient strains were further studied in bioreactor cultures with d-glucose pulses in nitrogen sufficient or restricted conditions. When *pgi1*-deficient *S. cerevisiae* strain H4346 expressing the d-glucaric acid pathway was grown in pH regulated batch cultures at pH 5.5, 1.3 g d-glucaric acid L^−1^ (yield 0.12 ± 0.01 g d-glucaric acid [g d-glucose consumed]^−1^) were produced (H4346, Figs. 3, 4a). d-Glucaric acid was produced at a rate of approximately 7 mg L^−1^ h^−1^. Myo-inositol was detectable in the culture supernatant after 45 h (0.06 g L^−1^) and accumulated in proportion (10 ± 1%) to d-glucaric acid to a final concentration of 0.15 g L^−1^. Up to 0.7 g glycerol L^−1^ and 2.5 g acetate L^−1^ were also produced, but acetate was consumed when ethanol was not available as a carbon source. Only 4.7 ± 0.1 g biomass L^−1^ was produced from the 15.9 g d-fructose L^−1^, 14.7 g d-glucose L^−1^ and 6.1 g ethanol L^−1^ which were provided (yield of biomass on substrate ~ 0.17 g [g substrate consumed]^−1^).

The intracellular concentration of d-glucaric acid was constant at 10 ± 1 mg [g biomass]^−1^. Intracellular myo-inositol concentrations (6 ± 1 mg [g biomass]^−1^) were similar to those of d-glucaric acid, and the ratio of myo-inositol to d-glucaric acid in the cytoplasm was much higher than in the supernatant.

Methylene blue staining showed that 63 ± 2% of the cells were metabolically inactive after 70 h (Fig. 3). A high proportion of metabolically inactive cells was also observed for the *pgi1*-deficient parent strain without the d-glucaric acid pathway in flask cultures (58 ± 2%, after 43 h on SC medium with 12 g d-fructose L^−1^ and 0.5 g d-glucose L^−1^ and 92 ± 1% metabolically inactive cells with 12 g d-fructose L^−1^ and 3.4 g d-glucose L^−1^), indicating that the high level of metabolically inactive cells was a response to the relatively high concentration of d-glucose in the medium, not to d-glucaric acid production. Some cells adapted to the presence of d-glucose in the medium after ~ 90 h in the bioreactor and the proportion of metabolically inactive cells did not increase much after 70 h.

The *pgi1*-deficient d-glucaric acid strain H4346 produced 0.71 g d-glucaric acid L^−1^ after 160 h, with a yield of 0.23 g d-glucaric acid [g d-glucose consumed]^−1^, in nitrogen-restricted medium (Fig. 4b). There was 0.12 g myo-inositol L^−1^ also produced. No adaptation to the presence of d-glucose occurred, and this strain produced only 3.7 ± 0.1 g biomass L^−1^, with 91 ± 0.5% of cells metabolically inactive after 138 h. The concentration of intracellular d-glucaric acid (8 ± 2 mg [g biomass]^−1^) and myo-inositol (9 ± 4 mg [g biomass]^−1^) were similar to those observed in the nitrogen sufficient culture. Another *pgi1*-deficient d-glucaric acid strain with *INM1* integrated (H4350) followed a similar trend in d-glucaric acid production but produced less biomass and myo-inositol (Fig. 4b, lower panel).

To lower the d-glucose toxicity by fed-batch cultivation the *pgi1*-deficient d-glucaric acid strains, H4350 and H4346 were grown with d-glucose and d-fructose in the feed. d-Glucose concentration was maintained at less than 2.2 g L^−1^ during the first 120 h feeding, with d-fructose concentrations less than 1.5 g L^−1^ The strains produced 0.32–0.34 g d-glucaric acid L^−1^ after 117 h, with yield of 0.12 g d-glucaric acid [g d-glucose consumed]^−1^, but no further production after 117 h. The d-glucose concentration increased after 117 h, reaching concentrations of 3.7–4.5 g L^−1^ by 160 h (data not shown). Cell viability was measured at 138 h, with 63 ± 4% and 71 ± 4% metabolically inactive cells for strains H4350 and H4346, respectively.

### Polymeric substrates for production of d-glucaric acid

To evaluate use of cheap polymeric substrates for production of d-glucaric acid by the *pgi1*-deficient *S. cerevisiae* strains a commercial polysaccharide and α-cellulose were used as carbon sources and hydrolytic enzymes used for d-glucose release during d-glucaric acid production.

**Fig. 5:**
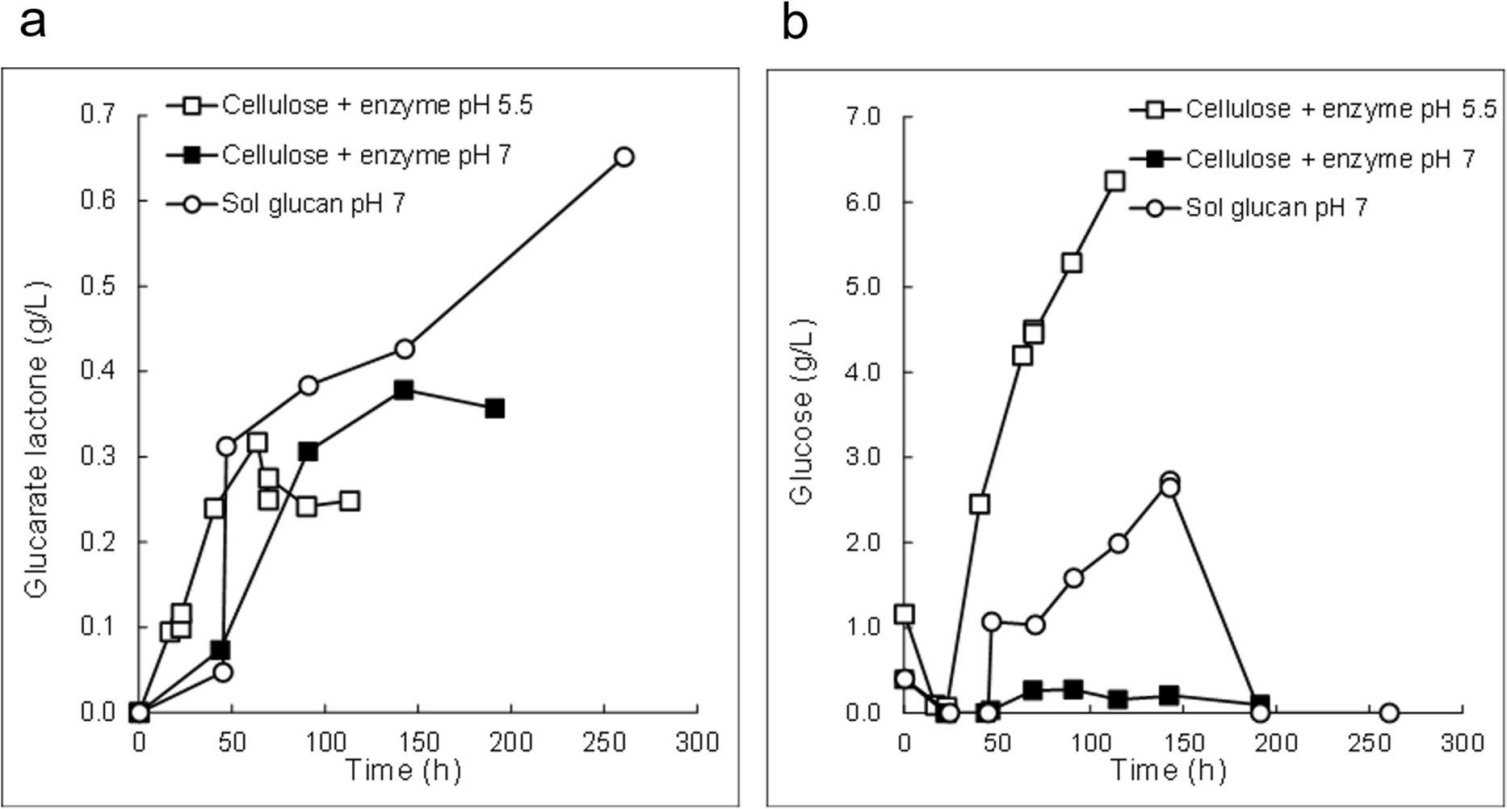
d-Glucaric acid production in bioreactor cultures with polymeric substrates. **a** d-Glucaric acid production. **b** d-Glucose release and consumption. Cellulose with hydrolytic enzyme in pH 5.5 (open square) or in pH 7 (filled square), commercial polysaccharide with hydrolytic enzyme (Sol glucan pH 7)

The d-glucose slowly released from the commercial polysaccharide was rapidly consumed and no d-glucose was detected in the culture media in shake flask cultures. In 100 h 12 g L^−1^ of d-glucose (as approximated from medium without cells) was released. To decrease production of biomass the amount of nitrogen provided was reduced. d-Fructose provided as a carbon source was consumed during the first 20 h. The *pgi1*-deficient, d-glucaric acid-pathway-expressing strains H4346 and H4351 produced 0.79 and 0.51 g L^−1^ d-glucaric acid within 100 h, respectively. Myo-inositol (~ 0.2 g L^−1^) and biomass were also formed. In bioreactors a maximum ~ 0.65 g d-glucaric acid L^−1^ was produced from the commercial polysaccharide, and 0.32 (pH 5.5) and 0.35 (pH 7) g d-glucaric acid L^−1^ was produced from α-cellulose within less than 250 h (Fig. 5a, lactone assay). In the cellulose pH 5.5 culture d-glucose started to accumulate from the beginning of the culture, and in the culture with the commercial polysaccharide after 50 h, whereas with cellulose pH 7 culture d-glucose remained low throughout the culture (Fig. 5b).

## Discussion

In *S. cerevisiae* the major flux of d-glucose is through glycolysis, and only a small fraction, approximately 1–4%, is channelled to the PPP, depending on strain and culture conditions (Blank et al. 2005; Fiaux et al. 2003; Gancedo and Lagunas 1973; Maaheimo et al. 2001; Nidelet et al. 2016). In the *pgi1*-deficient *S. cerevisiae* strains, the block to glycolysis leads to accumulation of glucose-6-phosphate (e.g. Ciriacy and Breitenbach 1979; Heux et al. 2008; Maitra 1971). Possibly, this glucose-6-phosphate accumulation could increase the flux of glucose-6-phosphate to myo-inositol and further to d-glucaric acid. Indeed, the yield of d-glucaric acid on d-glucose (0.12 g g^−1^) in the bioreactor with pulsed d-glucose was four-fold higher in our *pgi1*-deficient strains compared to the value in a strain with intact Pgi1p reported by Gupta et al. (2016) and was further increased to 0.23 g g^−1^ (seven fold improvement over Gupta et al. (2016)) by restricting the nitrogen supply. Recently, Guo et al. reported a yield of 0.216 g g^−1^ (Guo et al. 2022), close to our best yield. However, considering that our strains accumulated inositol at 12–17% of the d-glucaric acid concentration, it is clear that the yield could be even higher if all myo-inositol would be converted to d-glucaric acid. In *S. cerevisiae*, the *PGI1* deletion alone increased the yield, compared to *E. coli* where the *zwf* deletion was also needed (Shiue et al. 2015). In yeast, deletion or downregulation of both *PGI1* and glucose-6-phosphate dehydrogenase encoding gene *ZWF1* has not been reported, but during preparation of this work Zhao et al. (2023) reported that downregulation of *ZWF1* in *S. cerevisiae* improved d-glucaric acid titer by 22.4%.

In volumetric terms our *pgi1*-deficient strains produced about half of the d-glucaric acid concentration reported by Gupta et al. (2016) in shake flasks (average 0.26 compared to 0.54 g L^−1^), as confirmed by using ^13^C-labelled d-glucose. The titer was improved to 1.3 g L^−1^ in the bioreactor by providing the d-glucose in pulses, which was higher than reported by Gupta et al. (2016), but lower compared to recently reported volumetric titers of up to 9.5 g L^−1^ (Table 1). Also, the highest yield was observed with restricted nitrogen supply, and in this condition the volumetric concentration was only 0.71 g L^−1^. Interestingly, our parent strains with intact Pgi1p produced only ~ 30 mg L^−1^ of d-glucaric acid as measured with GC–MS. This in line with the results of Liu et al. (2016) who found that *P. pastoris* produced 108 mg L^−1^ d-glucaric acid on d-glucose, but much lower compared to other studies (Table 1). In our strains the *OPI1* gene was intact. We hypothesized that expression of *INO1* under a constitutive promoter is comparable to the effect of *OPI1* deletion because Opi1p is reported to regulate *INO1* at the transcriptional level (Henry et al. 2014; Ye et al. 2013). However, possibly the *OPI1* deletion could improve d-glucaric acid production also in the *pgi1*-deficient strains by a still unknown mechanism. In addition, differences in the strain background, pathway genes, and culture conditions, e.g. aeration and medium composition, may contribute to the differences in D-glucaric acid amounts produced.

The *S. cerevisiae pgi1*-deficient strains do not tolerate d-glucose concentration above 2 g L^−1^, potentially decreasing their viability, and suitability to larger scale processes. Our fed-batch cultures did not drastically improve viability and more optimised feeding strategies would be needed. The viability was better on polymeric substrate α-cellulose where hydrolytic enzymes were used to release d-glucose, but the rate of d-glucose release would require further optimization to increase d-glucaric acid production. Recently, consolidated bioprocess (CBP) with concomitant sugar release and conversion to products has been developed for d-glucaric acid production (Fang et al. 2022, 2023; Li et al. 2021). This could be an option for the *pgi1*-deficient strains.

d-Glucaric acid production with *S. cerevisiae* has developed rapidly during recent years, especially considering volumetric titers (Table1). New myo-inositol oxygenases (Marques et al. 2020), and ways for improved viability (Guo et al. 2022) would be highly interesting to test in the *pgi1*-deficient *S. cerevisiae*. Also, uncoupling growth and production, or regulating the Pgi1p amount by synthetic promoters, gene switches, and/or degradation approaches like those implemented in *E. coli* (Brockman and Prather 2015; Gupta et al. 2017; Hou et al. 2020; Qu et al. 2018) could improve d-glucaric acid production and viability in the *pgi1*-deficient *S. cerevisiae*.

## Data Availability

The datasets generated during and/or analysed during the current study are available from the corresponding author on reasonable request.
